# BTLA Expression and Function Are Impaired on SLE B Cells

**DOI:** 10.3389/fimmu.2021.667991

**Published:** 2021-04-22

**Authors:** Annika Wiedemann, Marie Lettau, Sarah Y. Weißenberg, Ana-Luisa Stefanski, Eva-Vanessa Schrezenmeier, Hector Rincon-Arevalo, Karin Reiter, Tobias Alexander, Falk Hiepe, Andreia C. Lino, Thomas Dörner

**Affiliations:** ^1^ Department of Rheumatology and Clinical Immunology, Charité- Universitätsmedizin Berlin, Berlin, Germany; ^2^ German Rheumatism Research Center Berlin (DRFZ), Berlin, Germany; ^3^ Department of Nephrology and Intensive Medical Care, BIH Charité- Universitätsmedizin Berlin, Berlin, Germany; ^4^ Grupo de Inmunología Celular e Inmunogenética, Facultad de Medicina, Instituto de Investigaciones Médicas, Universidad de Antioquia UdeA, Medellín, Colombia

**Keywords:** BTLA/CD272, SLE, B cells, SYK, plasmacytosis

## Abstract

B- and T-lymphocyte attenuator (BTLA/CD272) is an inhibitory checkpoint molecule expressed on T and B cells. Prior studies reported defective function of BTLA by T cells in patients with systemic lupus erythematosus (SLE), whereas nothing is known about its role on B cells in SLE, a disease with various B cell abnormalities. Peripheral blood mononuclear cells (PBMCs) from 23 healthy donors (HD) and 34 SLE patients were stained for BTLA and its expression on B cells was assessed. PBMCs or CD27^-^IgD^+^ naive B cells were stimulated together with an activating anti-BTLA antibody or an inhibitor of spleen tyrosine kinase (SYK) and differentiation as well as the expression of activation markers CD71, PD-1 and CD86 were analyzed. Our phenotypic and functional studies revealed reduced BTLA expression on CD27^-^IgD^+^ naïve B cells from SLE patients (p=0.0017) related to anti-dsDNA antibody titers (p=0.0394) and SIGLEC-1/CD169 expression on monocytes (p=0.0196), a type I interferon marker related to disease activity. BTLA engagement was found to control CpG/TLR9 activation limiting plasmablast (p=0.0156) and B cell memory induction (p=0.0078) in normal B cells in contrast to other B cell activation pathways (CD40, BCR). These BTLA functions were impaired in SLE B cells. Inhibition of SYK was found to mimic the effects of BTLA activity *in vitro*. Thus, is it possible that reduced BTLA expression and function of CD27-IgD+ antigen- and T cell-inexperienced SLE B cells could be overcome by SYK inhibition which should be tested in future studies as potential therapeutic principle.

## Introduction

B- and T-lymphocyte attenuator (BTLA/CD272) is an inhibitory checkpoint molecule that is constitutively expressed on B cells (1, 2) and permanently associated with the B cell receptor (BCR) (3). The type I transmembrane glycoprotein of the immunoglobulin (Ig) superfamily contains three tyrosine residues within immunoreceptor-tyrosine-inhibitory motifs (ITIMs) and immunoreceptor-tyrosine-switch-motif (ITSM) (4). Upon activation, tyrosine residues are phosphorylated and recruit the phosphatase Src-homology-region-2-domain-containing phosphatase-1 (SHP-1), which dephosphorylates downstream kinases (4). The ligand of BTLA, herpesvirus entry mediator (HVEM) is expressed on resting T cells, naïve and memory B cells (mBC) in the peripheral blood and belongs to the tumor necrosis factor receptor (TNFR) family (5). Functionally, BTLA engagement reduces proliferation, cytokine production and cytolytic function of T cells (4, 6, 7).

BTLA^-/-^ mice show a breakdown of B cell tolerance with polyclonal B and T cell activation (8) and lack of BTLA accelerates a lupus-like phenotype (9). Systemic lupus erythematosus (SLE) is an autoantibody-mediated autoimmune disease characterized by a breach of immune tolerance resulting in substantial B and T cell abnormalities. Characteristically, abundance of autoantigens (10) in SLE together with enhanced plasmacytosis related to disease activity reflect constant activation of (auto)immune cells with simultaneous predominance of post-activated B and T cells (11–13). In humans, a functional polymorphism of BTLA is associated with susceptibility for rheumatoid arthritis (RA) (14), whereas no differences in alleles, genotypes and haplotypes of the BTLA gene were found between SLE patients and healthy donors (HD) (15). There are different findings regarding BTLA expressed on lupus T cells (6, 16), even though SLE T cells exhibited abnormal BTLA function (6). In HD, BTLA is highly expressed on naïve B cells and regulates BCR responses (1, 3) but data regarding BTLA expression on SLE B cells and its potential role in autoimmunity are lacking.

## Materials and Methods

### Donors

EDTA-anticoagulated blood was drawn from 23 HD (age 36 ± 12y (mean ± standard deviation SD), range 22-62y, 65% female) and 34 SLE patients (age 38 ± 11y, range 23-64y, 94% female). Donor demographics including patients’ medications can be found in the supplementary material (**Table S1**). The study was approved by the local ethics committee of Charité Universitätsmedizin Berlin and written consent was obtained from all patients.

### Isolation of Mononuclear Cells

PBMCs were isolated as described previously (17).

### Whole Blood Stainings for SIGLEC-1

Lysis of EDTA-anticoagulated blood and staining for SIGLEC-1 expression on CD14^+^ monocytes was performed as previously described (18).

### Stainings for Flow Cytometry

0.5-2x10^6^ PBMCs were stained for 15 min at 4°C with different combinations of antibodies (**Table S2**) and washed before acquisition. 4,6-Diamidine-2-Phenylindole (DAPI) (Molecular Probes, Eugene, USA) or LIVE/DEAD Fixable Blue Dead Cell Stain Kit (ThermoFisher Scientific, Waltham, USA) was used to identify dead cells according to the manufacturer’s protocol. Cells were acquired on a FACS Canto II or LSR Fortessa X-20 flow cytometer (BD Biosciences, Heidelberg, Germany) (**Figure S1A**) (19). For quality control, CS&T Beads (BD Biosciences), SHPERO Calibration Particles (BD Biosciences) and in some experiments, application settings have been used to obtain reproducible median fluorescence intensities (MFIs). The isotype control of BTLA was measured in a fluorescence minus one (FMO) control approach.

### Magnetic-Activated Cell Sorting and Fluorescence-Activated Cell Sorting

B cells were purified using B cell isolation kit II according to the manufacturer’s instructions (Miltenyi, Bergisch Gladbach, Germany), stained and CD19^+^CD20^+^CD27^-^IgD^+^ B cells were sorted with a FACS Aria I or FACS Aria II cell sorter (BD Biosciences) (**Figure S1B**).

### Cell Culture

PBMCs or sorted naïve B cells were rested for 30 min at 37°C in medium (RPMI 1640/10% FBS/1%Pen/Strep, all ThermoFisher). 96-well cell culture plates were coated for 2 hours at 37°C with 10 µg/ml anti-BTLA antibody (clone MIH26, Biolegend, San Diego, USA) or 10 µg/ml isotype control (mouse IgG2aκ, Biolegend) and washed two times with PBS. 1x10^6^ PBMCs or 3-5x10^4^ naïve B cells were seeded per well and pre-incubated with coated antibodies for 30 min. Naïve B cells were stimulated with 0.02 µg/ml IL-2 (Miltenyi), 0.02 µg/ml IL-10 (Miltenyi), 0.5 µg/ml aBCR F(ab)_2_ IgM/IgA/IgG (aBCR, Jackson ImmunoResearch, Ely, UK), 2.5 µg/ml CpG ODN 2006 (Miltenyi) and 0.5 µg/ml previously crosslinked CD40L (Miltenyi) or medium as control at 37°C and 5% CO2 in a humidified incubator. PBMCs were cultured under the same conditions and with the same stimulation cocktail mentioned above or with CpG or CD40L alone. In some experiments, 10 µM SYK inhibitor entospletinib (GS-9973, SelleckChem, Munich, Germany) dissolved in DMSO or DMSO (Sigma Aldrich, St. Louis, MO, USA) alone as a control was added to the culture. After five days, cells were harvested and subjected to staining. Concentrations were used according to prior titration experiments.

### Short-Term Stimulation

PBMCs were rested for 30 min in RPMI at 37°C after isolation and stimulated with 19.5 µg/ml aBCR F(ab)_2_ IgM/IgA/IgG or RPMI for 5 min. Cells were then lysed/fixed (BD Lyse/Fix Buffer, BD Biosciences) and subsequently permeabilized with Perm Buffer II (BD Biosciences) according to the manufacturer’s instructions. Intracellular staining of pSYK Y^352^ and markers to identify respective subsets was carried out for 1 h at room temperature and cells were acquired on a BD LSR Fortessa X-20.

### Data and Statistical Analysis

Flow cytometric data was analyzed with FlowJo version 10 (FlowJo, BD Biosciences. Statistical analysis was performed with GraphPad Prism 6 or 8 software (Graphpad Software). Significance was tested by Wilcoxon signed rank test for paired comparisons and Mann Whitney U test for comparison of unpaired samples. Correlation was assessed by Spearman’s rank correlation. Differences with a P value < 0.05 were considered statistically significant.

## Results

### Reduced BTLA Expression Is Characteristic of Naïve SLE B Cells

**Figure 1:**
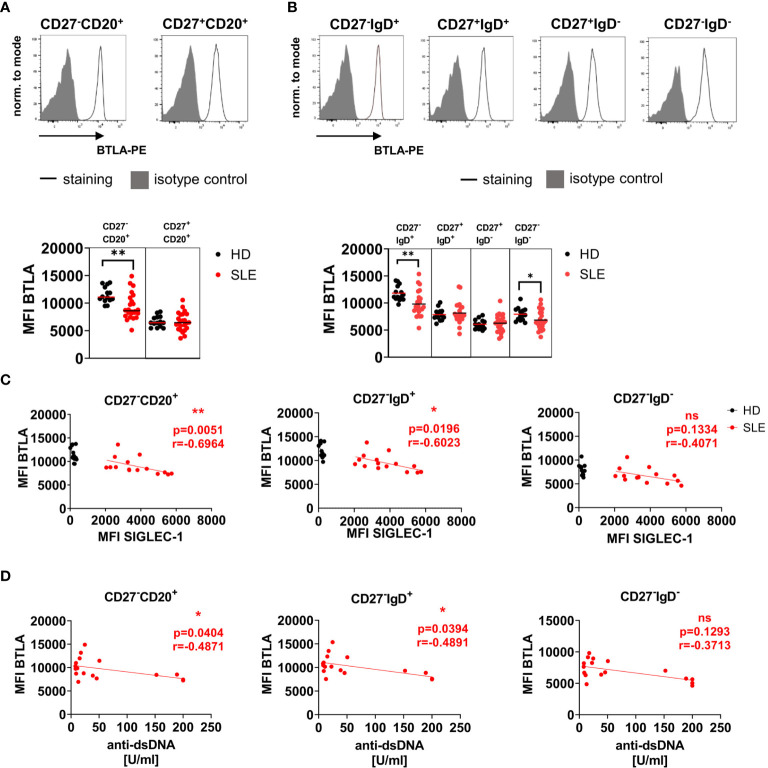
BTLA surface expression is reduced on naïve SLE B cells and inversely correlates with SIGLEC-1 (CD169) expression and anti-dsDNA antibody titers in SLE patients. **(A, B)** Representative histograms of BTLA expression and isotype control on B cell subsets in HD defined by CD27 and CD20 **(A)** and CD27 and IgD **(B)** derived from peripheral blood and summary data (MFI) for SLE patients and HD. Bars represent median. HD n=14, SLE n=24, Mann-Whitney U Test. **(C)** Spearman’s rank correlation of BTLA expression (MFI) on CD27^-^CD20^+^, CD27^-^IgD^+^ and CD27^-^IgD^-^ B cells with SIGLEC-1 expression on CD14+ monocytes in patients with SIGLEC-1 MFI >2000, HD are shown as control (HD n=12, SLE n=15). **(D)** Spearman’s rank correlation of BTLA surface expression (MFI) on CD27^-^CD20^+^, CD27^-^IgD^+^ and CD27^-^IgD^-^ B cells with anti-dsDNA antibody titers (U/ml, n=18). *p < 0.05, **p < 0.01, ns means not significant.

BTLA expression was compared between B cell subsets from HD and SLE patients (**Figure 1**). BTLA was higher expressed on CD27**^-^**CD20**^+^** conventional naïve B cells compared to CD27^+^CD20^+^ conventional mBC in all donors (**Figure 1A**, gating strategy **Figure S1A**). CD27^-^CD20^+^ conventional naïve B cells of SLE patients displayed a significantly lower BTLA expression compared to HD (p=0.0016). This lower BTLA expression was detected for both CD27^-^IgD^+^ pre-switch naïve and CD27^-^IgD^-^ double-negative (DN, atypical) SLE mBCs compared to HD (**Figure 1B**, CD27^-^IgD^+^ p=0.0017, CD27^-^IgD^-^ p=0.0248), while CD27^+^ conventional mBC expressed similar levels of BTLA in SLE patients and HD. CD27^++^CD38^++^ plasma cells (PC) carried a substantially lower BTLA surface expression compared to naïve B cells similar to those of memory B cells (HD range MFI 5025-10451, SLE range 4021 – 10528) but the expression did not differ between SLE patients and HD (**Figure S2A**, p>0.05).

### BTLA Expression on Naïve Pre-Switch SLE B Cells Correlates Inversely With SIGLEC-1 Expression, a Marker for Type I Interferon Signature and Anti-dsDNA Antibody Titers

We further analyzed if BTLA expression on B cell subsets correlated with SIGLEC-1 (CD169) expression on monocytes, a type I interferon signature marker (20) and anti-double-stranded DNA (dsDNA) antibodies (**Figures 1C, D**). Among 23 SLE patients, two were SIGLEC-1 negative (MFI <500), while the others expressed moderate to high levels of SIGLEC-1 (range SIGLEC-1 MFI 320-5743). All HD were SIGLEC-1 negative (range SIGLEC-1 MFI 0-374). In SLE patients with a high interferon signature (MFI SIGLEC-1 >2000), the expression of BTLA on CD27^-^CD20^+^ and CD27^-^IgD^+^ pre-switch naïve B cells correlated inversely with SIGLEC-1 expression on monocytes (CD27^-^CD20^+^ p=0.0051, r=-0.6964, CD27^-^IgD^+^ p=0.0196, r=-0.6023, **Figure 1C**) as well as with anti-dsDNA titers (CD27^-^CD20^+^ p=0.0404, r=-0.4871, CD27^-^IgD^+^ p=0.0394, r=-0.4891, **Figure 1D**). There was no correlation between both parameters and BTLA expression on CD27^-^IgD^-^ DN (atypical) B cells (**Figures 1C, D**). BTLA expression on any of the B cells subsets did not correlate with lupus activity in patients with moderate to high disease activity (SLEDAI ≥4, **Figure S2B**).

### Activation of BTLA Reduces Differentiation of B Cells Into Plasmablasts in Healthy Controls

**Figure 2:**
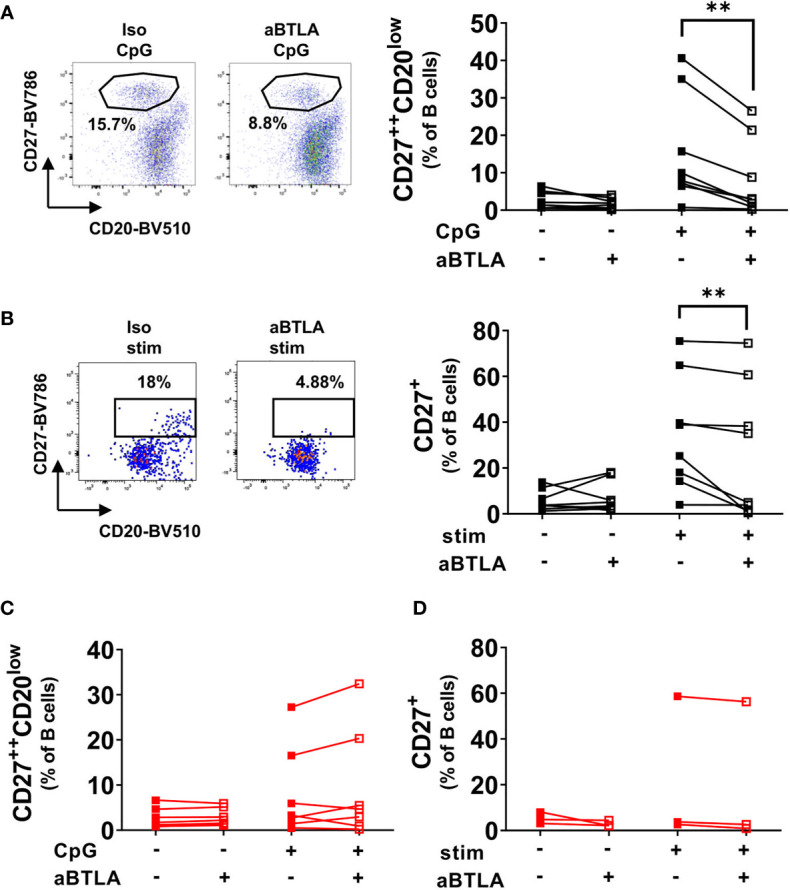
BTLA activation inhibits differentiation of HD B cells but not SLE B cells. **(A)** HD PBMCs were stimulated with CpG for five days in the presence of an activating anti-BTLA antibody or isotype control. Representative dotplots and cumulative data of plasmablast frequencies among CD19^+^CD20^+/-^ B cells. HD n=8. **(B)** Sorted CD27-IgD+ naïve HD B cells were stimulated with a combination of IL-2, IL-10, aBCR, CpG and CD40L for five days at 37°C and 5% CO_2_ in the presence of an activating anti-BTLA antibody or isotype control. Representative dot plots of CD19^+^CD20^+/-^ B cells and cumulative data of the frequency of CD27^+^ cells among CD19^+^CD20^+/-^ B cells. HD n=7. **(C)** SLE PBMCs were stimulated as in A). Representative dot plots of CD19^+^CD20^+/-^ B cells with plasmablast frequencies and cumulative data. n=7. **(D)** Sorted CD27^-^IgD^+^ naïve SLE B cells were stimulated as in B). Representative dot plots of CD19^+^CD20^+/-^ B cells and cumulative data of the frequency of CD27^+^ cells among CD19^+^CD20^+/-^ B cells. n=3. Statistical significance was assessed by using Wilcoxon signed rank test. **p < 0.01.

Functional studies addressed consequences of BTLA engagement on the differentiation of B cells into PB upon various stimulation conditions. After TLR9 stimulation in peripheral blood mononuclear cell (PBMC) cultures, we observed a substantial increase of CD27^++^CD20^low^ PB in HD (**Figure 2A**, medium control: 2.6 ± 2.3% (mean ± SD), CpG control: 15.4 ± 14.5%). Most noteworthy, pre-incubation with a functionally active anti-BTLA antibody resulted in a markedly diminished PB induction of HD B cells after stimulation (p=0.0156, 8.2 ± 10.2%), whereas no such effect was observed after CD40 stimulation alone or combined stimulation of BCR, CD40 and TLR9 (**Figure S3A**). This indicates that BTLA is a critical checkpoint molecule counteracting TLR9-dependent differentiation of B cells into PC.

### Expression of Activation Markers Is Not Affected by BTLA Treatment

Subsequently, we addressed whether markers of activation were altered by BTLA activation. We analyzed CD71 as a marker of proliferation, the costimulatory molecule CD86 as well as the co-inhibitory molecule PD-1 as activation markers at day five after stimulation with and without prior BTLA engagement (**Figures S3C–E**). All markers studied were upregulated in HD upon stimulation but pre-treatment with anti-BTLA did not alter the expression of these markers (p>0.05).

### BTLA Activation Inhibits Differentiation of Naïve B Cells From Healthy Controls *In Vitro*

Next, we wondered whether differentiation of naïve B cells upon stimulation with IL-2, IL-10, CpG, anti-BCR (aBCR) F(ab)_2_ IgM/IgG/IgA and CD40L for five days would be affected by BTLA activation, since BTLA expression was maximal on these cells at baseline compared to other B cell subsets (**Figures 1A, B**). Indeed, activation of BTLA reduced differentiation induced by stimulation of HD naïve B cells into CD27^+^ B cells (p=0.0078, **Figure 2B**). We conclude, that BTLA represents a checkpoint molecule that controls differentiation of naïve B cells into mBC.

### Activation of BTLA Does Not Inhibit or Reduce Differentiation of B Cells in SLE

Subsequently, PBMCs and sorted CD27^-^IgD^+^ naïve B cells of SLE patients were stimulated under the same conditions as HD cells (**Figures 2C, D**, **Figure S3B**). Interestingly and in striking contrast to control B cells, SLE B cells showed lower differentiation rates into PBs (CpG control: 8.2 ± 9.9%; CpG aBTLA: 9.6 ± 12.1%) and CD27^+^ B cells (stim control: 21.7 ± 31%; stim aBTLA: 19.9 ± 31.5%), and no changes were found under BTLA activation as shown by the resulting frequencies of PBs or CD27^+^ B cells (p=0.2969; p=0.2500). This observation was consistent with findings of isolated or combined stimuli (**Figure S3B**). The data suggest that differentiation of SLE B cells in contrast to HD are not controlled by BTLA.

### Inhibition of SYK Reduces Differentiation of B Cells *In Vitro*

**Figure 3:**
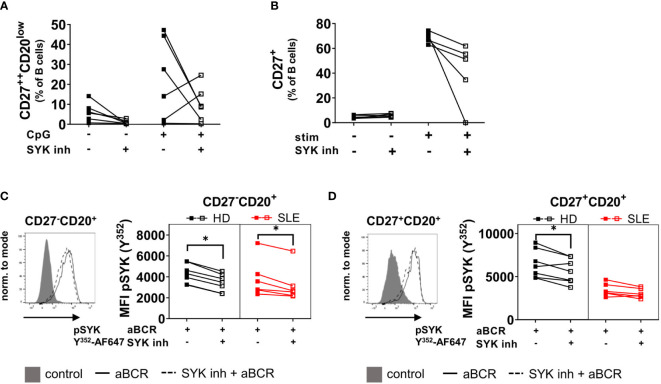
Inhibition of SYK results in reduced differentiation and activation of PBMC from HD and reduced phosphorylation of pSYK in pre-switch CD27-CD20+ B cells from HD and SLE patients. **(A)** HD PBMCs were stimulated with CpG for five days together with the SYK inhibitor entospletinib or DMSO as a control. Resulting frequencies of CD27^++^CD20^low^ plasmablasts among CD19^+^CD20^+/-^ B cells and cumulative data. HD n=6. **(B)** Sorted CD27^-^IgD^+^ naïve HD B cells were stimulated with a combination of IL-2, IL-10, CpG, aBCR and CD40L for five days at 37°C and 5% CO_2_ with or without the SYK inhibitor entospletinib. Cumulative data of the frequency of CD27^+^ cells among CD19^+^CD20^+/-^ B cells. HD n=5. **(C, D)** SLE and HD PBMCs were stimulated for 5 min with aBCR and stained for pSYK Y^352^ in combination with markers to identify CD27^-^CD20^+^ **(C)** and CD27^+^CD20^+^ **(D)** B cell subsets. A representative histogram and the MFI of pSYK Y^352^ is shown. HD n=7, SLE n=6. Wilcoxon signed rank test. *p < 0.05.

BTLA is constitutively associated with the BCR and recruits the phosphatase SHP-1 to the BCR complex upon activation which dephosphorylates SYK and thus counter regulates activation (3, 4). In addition, SYK plays an essential role in CpG-induced activation and differentiation (21) as also reported recently by our group (11). Therefore, we wondered if inhibition of SYK would lead to similar functional consequences of BTLA engagement during B cell activation. Indeed, inhibition of SYK led to a reduction of PB and CD27^+^ B cell formation of activated PBMC and naïve B cells from controls (**Figures 3A, B**) mimicking the effects seen for BTLA activation (**Figures 2A, B**).

### BCR-Induced Phosphorylation of SYK Cannot Be Inhibited in SLE Memory B Cells

Since short-term stimulation of the BCR leads to phosphorylation of SYK (11), we wanted to assess if SYK inhibition can reduce SYK phosphorylation in SLE and HD B cells as a first step of cell activation (**Figures 3C, D**). Staining of pSYK Y^352^ revealed reduced phosphorylation after pre-incubation with the SYK inhibitor entospletinib in CD27^-^CD20^+^ naïve SLE and HD B cells (SLE p=0.0313, HD p=0.0156, **Figure 3C**). CD27^+^CD20^+^ conventional memory B cells from SLE patients displayed lower responsiveness to BCR stimulation than HD (11) and the SYK inhibitor did not affect SYK phosphorylation in SLE memory B cells (p>0.05, **Figure 3D**). In HD mBC, entospletinib reduced SYK phosphorylation (p=0.0313). These data suggests that BTLA and SYK pathways may have overlapping activity.

## Discussion

We here describe BTLA expression on HD B cell subsets in comparison to those of SLE patients and functional *in vitro* effects of BTLA activation. While HD naïve B cells express higher levels of BTLA than mBC (1) we show that SLE naïve B cells exhibit significantly reduced expression of BTLA compared to HD naïve B cells, possibly leading to disturbances during B cell activation. Consistently, BTLA expression correlated inversely with SIGLEC-1 expression on monocytes as a type I IFN marker and anti-dsDNA antibody titers. Thus, a lower BTLA expression on naïve SLE B cells was related to a more pronounced IFN signature. Whether this is an intrinsic or a functional consequence remains unclear.

Activation of BTLA by a monoclonal antibody with known intrinsic activity did not alter the expression of markers for activation and B cell proliferation as described before (22). In BTLA-deficient B cells, slightly augmented responses to stimulation by anti-IgM were described (23). Here, we found a prominent effect of BTLA activation during CpG-induced differentiation of B cells to PB and differentiation from CD27^-^ to CD27^+^ B cells after combined stimulation of BCR, CD40 and TLR9. While HD B cells displayed reduced differentiation after anti-BTLA treatment, this effect was absent in SLE B cells suggesting a defective checkpoint of B cell differentiation. In this regard, studies in SLE patients reported that peripheral PB originate not only from memory but also naïve B cells (24) consistent with defective checkpoints for memory as well as naïve B cells. Stimulation with CpG induced clustering of BTLA with the BCR in 55% of B cells similar to stimulation with anti-IgM, suggesting a role for BTLA during CpG-induced stimulation next to BCR signaling (25). Since we studied plasmablast differentiation in PBMC cultures, non-B cells expressing BTLA could be affected by anti-BTLA and could act indirectly on B stimulation. However, the impact seems to be different between HD and SLE patients although additional studies on purified B cells would be required.

SLE B cells are described to be in a post-activated state (11, 12) affecting naïve and memory B cells in contrast to Sjögren’s and rheumatoid patients where only memory B cells are affected (11), and do not respond as well to stimulation as HD B cells. This is related to enhanced protein tyrosine/serine/threonine phosphatase activity leading to reduced overall phosphorylation upon stimulation (11). We observed similar effects for BTLA activation able to engage phosphatase Src-homology-region-2-domain-containing phosphatase-1 (SHP-1) and SYK inhibition where the net result of BTLA leading to increased phosphatase activity including control of Syk phosphorylation could explain the current findings. How the interaction of these two pathways is interrelated, is subject of further research.

Since we included mainly active SLE patients, post-activated SLE B cells did not react well to stimulation. Additionally, all of the patients received medication due to active disease, including a majority receiving hydroxychloroquine. Prior studies (11, 12), however, could also identify anergic post-activated naïve and memory B cells in untreated, new onset SLE as intrinsic characteristic independent of treatment. Thus, studies of patients carrying a broader spectrum of lupus activity and possibly also without receiving any medication might be important to further elucidate BTLA function in SLE.

In summary, reduced BTLA expression and lack of inhibition during differentiation into mBC and PB in SLE patients is consistent with an intrinsically abnormal checkpoint function of BTLA. This lack of immune control may explain enhanced plasmacytosis in patients originating from memory but also naïve SLE B cells. Inhibition of a key downstream phosphokinase, SYK, as a crossroad of BCR and TLR9 activation, may overcome this abnormal checkpoint of comprehensive B cell disturbances in SLE. Our data suggests that BTLA and SYK pathways may overlap with the possibility that SYK inhibitors may overcome abnormalities of BTLA in SLE. Even though, the SYK inhibitor fostamatinib has already demonstrated efficacy in another humoral autoimmune disease, immune thrombocytopenia (26), additional investigations are needed to better understand the mechanistic overlap between BTLA and SYK as potential new strategy for SLE.

## Data Availability Statement

The original contributions presented in the study are included in the article/**Supplementary Material** Further inquiries can be directed to the corresponding author.

## Ethics Statement

The studies involving human participants were reviewed and approved by Ethics Committee Charité Universitätsmedizin Berlin. The patients/participants provided their written informed consent to participate in this study.

## Author Contributions

AW, ACL, and TD planned the research and designed experiments. ML, E-VS, A-LS, TA and FH recruited donors. AW, ML, SW, E-VS, HR-A, and KR did the experiments and acquired and analyzed the data. AW wrote the first draft of the manuscript, ACL and TD revised the manuscript and provided advice. All authors contributed to the article and approved the submitted version.

## Funding

This study was funded by the DFG (DFG projects Do491/7-5, 10-1, 11-1, TRR130, LI3540/1-1). E-VS is supported by the Clinician Scientist Program by the Berlin Institute of Health. A-LS is supported by DGRh Research Initiative 2020. HR-A is supported by COLCIENCIAS scholarship No. 727.

## Conflict of Interest

The authors declare that the research was conducted in the absence of any commercial or financial relationships that could be construed as a potential conflict of interest.
