# Supplementary material for: BTLA Expression and Function Are Impaired on SLE B Cells
Source: Front Immunol. 2021 Apr 22;12:667991. doi: 10.3389/fimmu.2021.667991 (PMC8100666; doi:10.3389/fimmu.2021.667991)
Supplement: Supplementary file 1 [file DataSheet_1.pdf]

Supplementary Material

1 Supplementary Figures and Tables

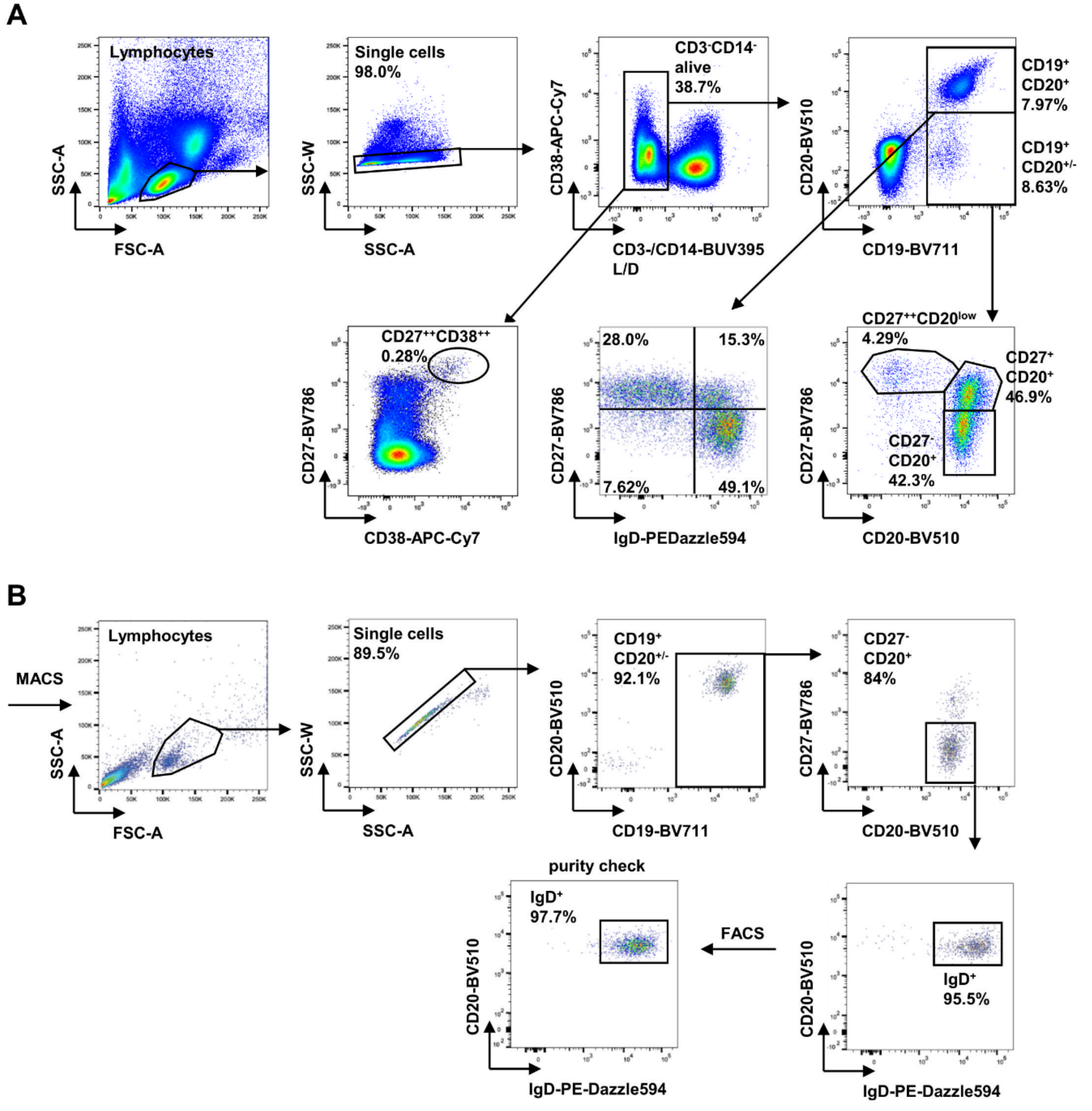

Figure S1. Gating of B cell subsets in peripheral blood. (**A**) Representative gating of PBMCs. Lymphocytes were gated by their scatter properties and doublets, CD3$^+$ T cells, CD14$^+$ monocytes and dead cells were excluded. CD19$^+$CD20$^{+/-}$ cells were subsequently categorized into CD27$^-$CD20$^+$ conventional naïve B cells, CD27$^+$CD20$^+$ conventional memory B cells and CD27^{++}CD20^{low} plasmablasts. For B cell subset gating by CD27 and IgD, CD19$^+$CD20$^+$ B cells were gated. Plasma cells were identified by high expression of CD27 and CD38 (CD27^{++}CD38^{++}) among CD3$^-$CD14$^-$

living cells. (**B**) After negative selection of B cells by magnetic-activated cell sorting (MACS), naïve B cells were sorted as $CD27^{-}IgD^{+}CD19^{+}CD20^{+}$ single lymphocytes by fluorescence-activated cell sorting (FACS) and the purity of the sorted population was validated.

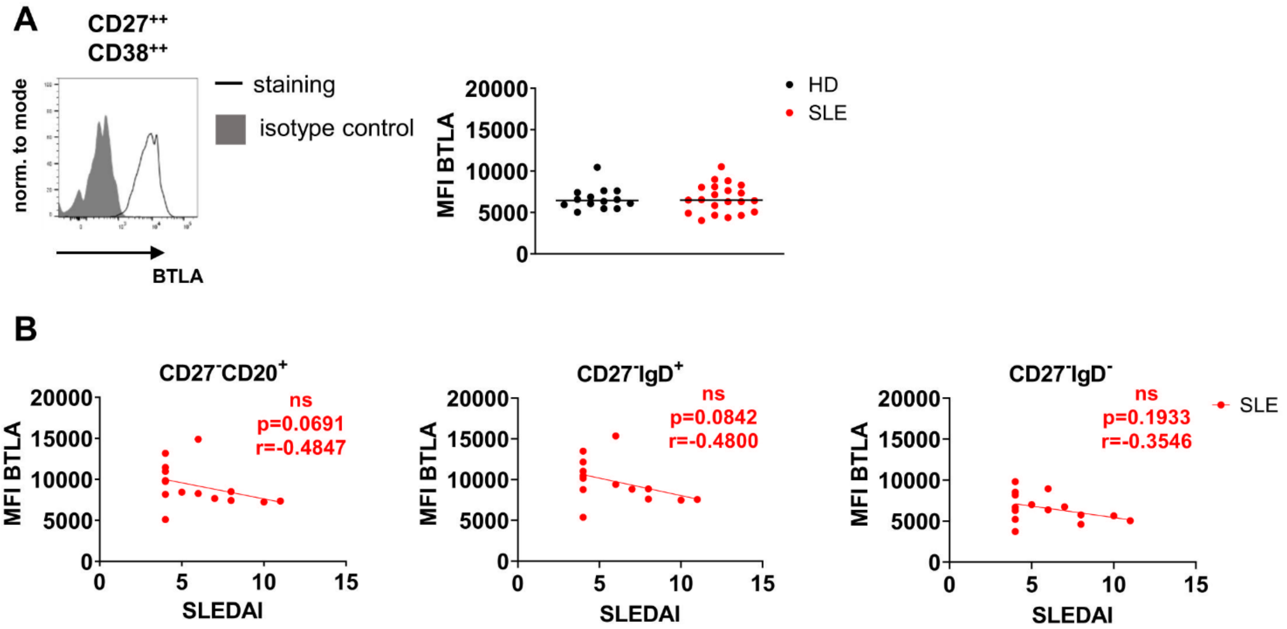

Figure S2. BTLA expression on human B cells. (**A**) Representative histogram of BTLA expression and isotype control on $CD27^{++}CD38^{++}$ PC and summary data (median fluorescence intensity MFI) for SLE patients and HD. Bar represents median, Mann-Whitney U Test, $p>0.05$. HD n=14, SLE n=21. (**B**) Spearman's rank correlation of BTLA expression on $CD27^{-}CD20^{+}$, $CD27^{-}IgD^{+}$ and $CD27^{-}IgD^{-}$ B cells with lupus activity assessed by SLEDAI (SLEDAI ≥4 n=15).

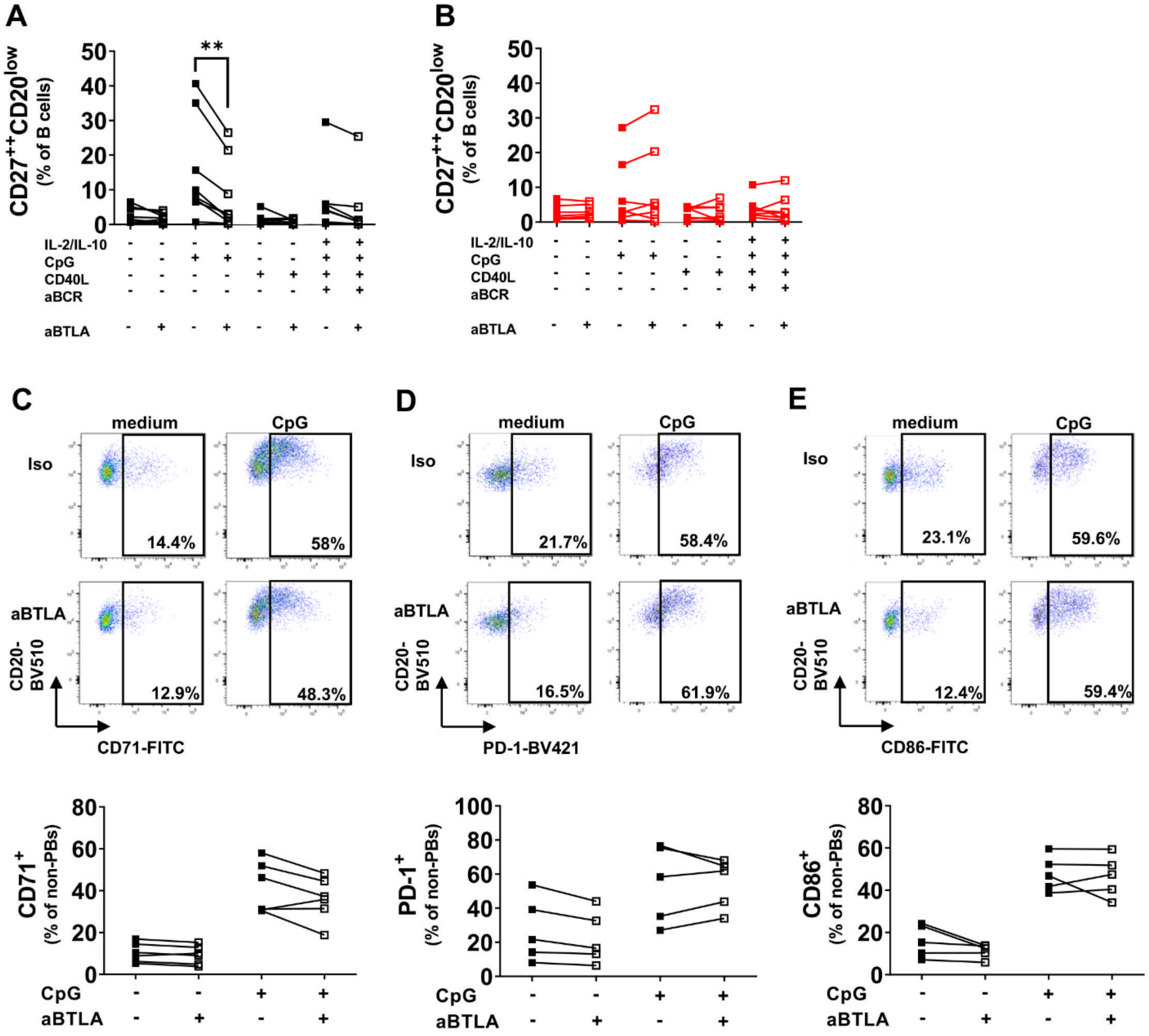

Figure S3. Plasmablast frequencies and expression of activation markers of HD and SLE after stimulation. (**A, B**) HD (**A**) and SLE (**B**) PMBCs have been stimulated with a combination of IL-2, IL-10, CpG, CD40L and aBCR or with CpG or CD40L alone for five days with or without prior addition of an anti-BTLA antibody with known intrinsic activity. Data from Figure 2A and C are included in this graph. HD n=8, SLE n=7. Wilcoxon signed rank test. (**C-E**) HD PBMCs were stimulated with CpG for five days in the presence of an activating anti-BTLA antibody or isotype control. Representative dotplots and cumulative data of CD71 (**C**), PD-1 (**D**) and CD86 (**E**) expression as frequency of on non-PBs (excluding $CD27^{++}CD20^{low}$ PBs) after stimulation with CpG. HD n=5-6.

Table S1. Donor demographics*.

Donor No	Gender	Age	Disease activity (SLEDAI)	Medication
HD1	F	40	-	-
HD2	F	43	-	-
HD3	F	25	-	-
HD4	F	29	-	-
HD5	F	26	-	-
HD6	F	25	-	-
HD7	F	unk	-	-
HD8	F	27	-	-
HD9	F	30	-	-
HD10	F	29	-	-
HD11	M	29	-	-
HD12	F	60	-	-
HD13	F	50	-	-
HD14	M	35	-	-
HD15	F	30	-	-
HD16	M	34	-	-
HD17	M	unk	-	-
HD18	F	27	-	-

**Donor No**	**Gender**	**Age**	**Disease activity (SLEDAI)**	**Medication**
HD19	M	32	-	-
HD20	M	62	-	-
HD21	F	40	-	-
HD22	M	57	-	-
HD23	M	22	-	-
SLE1	F	39	4	HCQ
SLE2	F	23	4	HCQ, Pred
SLE3	F	64	2	Pred
SLE4	F	33	4	HCQ
SLE5	F	30	8	MTX, Pred
SLE6	F	27	5	HCQ, MMF
SLE7	F	23	7	HCQ, MMF, Pred
SLE8	F	56	6	MMF
SLE9	F	48	4	Aza, Pred
SLE10	F	59	2	HCQ
SLE11	F	27	6	TNFi
SLE12	F	39	4	MTX, Pred, HCQ, MMF
SLE13	M	38	4	HCQ, MMF

Donor No	Gender	Age	Disease activity (SLEDAI)	Medication
SLE14	F	54	0	MMF
SLE15	F	29	10	Pred, MMF
SLE16	F	30	≤2	HCQ
SLE17	F	57	≤2	HCQ, MMF
SLE18	F	39	11	HCQ, Aza
SLE19	F	32	4	HCQ
SLE20	F	31	0	HCQ, Pred
SLE21	F	35	>8	HCQ, MMF, Pred
SLE22	F	43	2	HCQ
SLE23	F	26	0	HCQ, Aza, Pred
SLE24	F	42	4	MMF, Pred
SLE25	F	44	4	HCQ, MMF, Pred
SLE26	M	55	4	Belimumab
SLE27	F	25	10	HCQ, MMF, Pred, Belimumab
SLE28	F	33	4	Aza, Pred, Cloroquine
SLE29	F	40	16	Aza, HCQ, Pred
SLE30	F	44	16	HCQ, Pred
SLE31	F	30	17	HCQ, Pred

Donor No	Gender	Age	Disease activity (SLEDAI)	Medication
SLE32	F	43	>2	Pred, Aza, Belimumab
SLE33	F	24	10	Pred, HCQ, Ustekinumab
SLE34	F	35	4	Aza, Pred

*Azathioprine (Aza), female (f), hydroxychloroquine (HCQ), male (m), mycophenolatmofetil (MMF), methotrexate (MTX), systemic lupus erythematosus disease activity index (SLEDAI), TNF inhibitor (TNFi), unknown (unk).

Table S2. Antibodies used for flow cytometry and FACS*.

Target of antibody	Clone	Fluorochrome	Manufacturer
**CD3**	UCHT1	PacB, BUV395	BD Biosciences, Heidelberg, Germany
**CD14**	M5E2	PacB, BUV395	BD Biosciences, Heidelberg, Germany
	MOP9	APC-H7	BD Biosciences, Heidelberg, Germany
**CD19**	SJ25C1	PE-Cy7, BV711	BD Biosciences, Heidelberg, Germany
**CD20**	2H7	BV510	Biolegend, San Diego, USA
**CD27**	L128	APC, BV786, FITC	BD Biosciences, Heidelberg, Germany
**CD38**	HIT2	APC-Cy7	BD Biosciences, Heidelberg, Germany
**CD71**	OKT9	FITC	ThermoFisher Scientific, Waltham, MA, USA
**CD86**	2331(FUN-1)	FITC	BD Biosciences, Heidelberg, Germany
**BTLA**	MIH26	PE	Biolegend, San Diego, USA
**BTLA**	J168-540	APC	BD Biosciences, Heidelberg, Germany
**IgD**	IA6-2	PE-Dazzle594	Biolegend, San Diego, USA
**PD-1**	EH12.1	BV421	BD Biosciences, Heidelberg, Germany
**Siglec-1/CD169**	7-239	AF647	Biolegend, San Diego, USA
**pSYK Y^{352}**	17A/P-ZAP70	AF647	BD Biosciences, Heidelberg, Germany

*Alexa Fluor (AF), Allophycocyanine (APC), APC-Cyanine 7 (APC-Cy7), Brilliant Violet (BV), Brilliant Ultraviolet (BUV), Fluorescein isothiocyanate (FITC), Pacific Blue (PacB), Peridinin-chlorophyll protein cyanine 5.5 (PerCPCy5.5), Phycoerythrin (PE), PE-Cyanine 7 (PE-Cy7).
